# Disappeared supramolecular isomer reappears with perylene guest

**DOI:** 10.1107/S2052252520001451

**Published:** 2020-02-27

**Authors:** In-Hyeok Park, Atanu Dey, Kenta Sasaki, Masaaki Ohba, Shim Sung Lee, Jagadese J. Vittal

**Affiliations:** a Department of Chemistry, National University of Singapore, 3 Science Drive 3, 117543, Singapore; b Department of Chemistry, Kyushu University, 744 Motooka, Nishi-ku, Fukuoka 819-0395, Japan; c Department of Chemistry and Research Institute of Natural Science, Gyeongsang National University, Jinju 52828, Republic of Korea

**Keywords:** metal–organic frameworks, supramolecular isomers, disappearing polymorphism, crystal engineering, polymorphism, MOFs, coordination polymers, supramolecular isomerism

## Abstract

Among different types of polymorphism, disappearing polymorphism deals with the metastable kinetic form which can not be reproduced after its first isolation. In the world of coordination polymers (CPs) and metal–organic frameworks (MOFs), despite the fact that many types of supramolecular isomerism exist, we are unaware of disappearing supramolecular isomerism akin to disappearing polymorphism. This work reports a MOF with **dia** topology that could not be reproduced, but subsequent synthesis yielded another supramolecular isomer, a double-pillared-layer MOF. When perylene was added in the same reaction, the disappeared **dia** MOF reappeared with perylene as a guest in the channels. Interestingly, the photoluminescence of the **dia** MOF with a perylene guest is dominated by the emission of the guest molecule. The influence of guest molecules on the stabilization of the supramolecular isomers of a MOF opens up a strategy to access MOFs with different structures.

## Abstract 2

A sixfold interpenetrated metal–organic framework (MOF) with **dia** topology isolated in the first attempt could not be reproduced; subsequent synthesis yielded only the double-pillared-layer MOF. However, this ‘disappeared supramolecular isomer’ reappears in the presence of a perylene guest.

## 1. Introduction   

Polymorphism is a solid-state phenomenon in which the same chemical substance has more than one crystal structure (Brittain, 1999 ▸; Bernstein, 2008 ▸; Desiraju, 1997 ▸). Controlled and reproducible conditions are necessary to crystallize a particular polymorph of a compound, especially the metastable kinetic form. In many cases the metastable form fails to crystallize in numerous attempts after initial isolation; this has been termed ‘disappearing polymorphism’ (Dunitz & Bernstein, 1995 ▸). The subsequent isolation of these kinetics forms becomes elusive due to the crystallization of a more stable polymorph or poorly recorded crystallization conditions by the researchers (Dunitz & Bernstein, 1995 ▸; Bernstein & Henck, 1998 ▸; Lancaster *et al.*, 2007 ▸, 2011 ▸; Bučar *et al.*, 2015 ▸).

On the other hand, supramolecular isomerism, a phenomenon which is quite distinct from polymorphism, exists in coordination polymers (CPs) and metal–organic frameworks (MOFs) (Hennigar *et al.*, 1997 ▸; Moulton & Zaworotko, 2001 ▸). Since described by Zaworotko in 1997, a number of examples for various types of supramolecular isomerism have been reported (Zhang *et al.*, 2009 ▸; Park *et al.*, 2014*c* ▸, 2015 ▸, 2018 ▸, 2016 ▸, 2019 ▸; Karmakar *et al.*, 2017 ▸; Barnett *et al.*, 2002 ▸; Hu *et al.*, 2012 ▸; Blake *et al.*, 2001 ▸; Manna *et al.*, 2008 ▸; Poplaukhin & Tiekink, 2010 ▸; Panda *et al.*, 2013 ▸; Ju *et al.*, 2016 ▸). Interestingly, a ‘disappearing supramolecular isomer’ in CPs and MOFs is not known, unlike disappearing polymorphs in organic solids.

**Figure 1:**
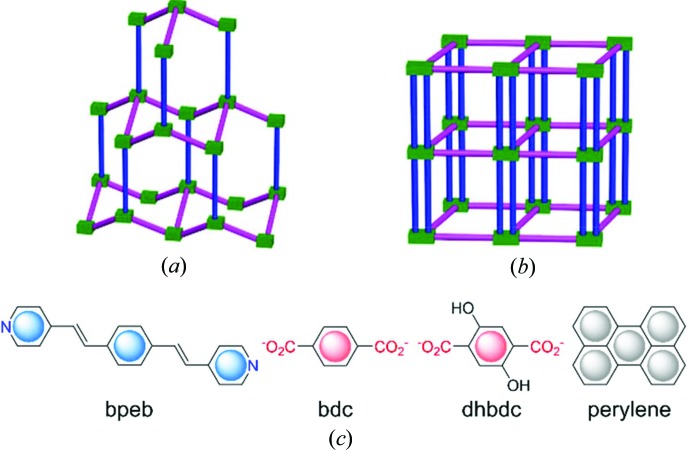
The two supramolecular isomers of [Zn(bpeb)(bdc)]. (*a*) Diamondoid structure. (*b*) Double-pillared-layer structure. Zn (green), bpeb (blue) and pink (bdc). (*c*) Ligand and guest structures.

We have recently encountered two supramolecular isomers in MOFs with the chemical formula [Zn(bpeb)(bdc)] {where bpeb = 1,4-bis[2-(4′-pyridyl)ethenyl]benzene and bdc = 1,4-benzenedicarboxylate; see Fig. 1 ▸} (Park *et al.*, 2016 ▸, 2014*b* ▸, 2018 ▸). They have diamondoid (**dia**) and double-pillared-layer structures [Figs. 1 ▸(*a*) and 1 ▸(*b*)]. Further, two more isomers are possible for the double-pillared-layer compounds arising from the bdc ligand coordination modes in the [Zn(bdc)] layers (Park *et al.*, 2018 ▸).

## 2. Experimental   

### 4.1. General   

Powder X-ray diffraction (PXRD) data were recorded on a D5005 Siemens X-ray diffractometer with graphite monochromated Cu  *K*α radiation (λ = 1.54056 Å) at room temperature (298 K). ^1^H-NMR spectra were recorded on a 300 MHz Bruker Advance 300 FT-NMR spectrometer by calibrating the residual solvent as the reference in DMSO-*d*_6_ solution. Solid-state diffuse reflectance UV–vis spectra were measured on a UV-2450 Shimadzu UV–vis spectrometer equipped with an integrating sphere and barium sulfate as the reflecting reference. The solid-state fluorescence measurements were made with a Horiba Fluorolog using a solid-state sample holder (excitation wavelength at 350 nm). Thermogravimetric analysis was performed using the Discovery TGA TA instrument under nitrogen gas flow with a heating rate of 5°C min^−1^ and the data were analysed using *TRIOS* (version 3.1, TA Instruments). The elemental analysis was performed using the ElementarVario Micro Cube instrument at the Elemental Analysis Lab, CMMAC, Department of Chemistry, National University of Singapore. Confocal microscopy imaging of two-photon-excited MOF crystals was achieved using the Leica TCS SP5 X at the Department of Biological Sciences, National University of Singapore.

Photoluminescence (PL) and two-photon photoluminescence (2PPL) measurements of MOF samples were conducted using a home-built optical setup under microscope (Nikon, Eclipse Ti) conditions. The excitation source is a titanium:sapphire oscillator generated laser with 140 fs pulses and 80 MHz repetition rate. A 10× objective lens (NA 0.3) was placed before the samples to focus the light beam. The MOF samples were placed on a piezo stage (PI P-545). The PL and 2PPL signals were accumulated by the same objective and reflected to the spectrometer by mirrors. The spectra were detected by a monochromator (Acton, Spectra Pro 2300i) assembled CCD (Princeton Instruments, Pixis 100B). For the PL measurement, the 800 nm incident laser light was tuned to 400 nm by a beta barium borate (BBO) crystal. The 400 nm beam was cleaned by a 400/10 nm band pass filter (Thorlabs, FB400-10) before illumination of the samples. The scattering of 400 nm light was blocked by a 450 long pass filter (Newport, 10LWF-450-B) before it entered the spectrometer. For the 2PPL measurement, the 800 nm incident laser light was purified by one 785 nm long pass filter (Semrock, LP02-785RU-25). The reflected incident light was cleared by a 785 short pass filter (Semrock, SP01-785RU-25).

### 4.2. Synthesis of [Zn(bpeb)(dhbdc)]·*x*DMA·*y*H_2_O (1)   

A mixture of bpeb (20.0 mg, 0.070 mmol), 2,5-dihydroxy-1,4,-benzenedicarboxylic acid (H_2_dhbdc) (13.8 mg, 0.070 mmol) and Zn(NO_3_)_2_·4H_2_O (18.4 mg, 0.070 mmol) dissolved in DMA (3.0 ml), DMSO (0.5 ml) and H_2_O (1.0 ml) was placed in a 10 ml glass tube, and then 0.1 ml of 0.1*M* NaOH solution was added. The tube was sealed and kept at 120°C for 48 h, followed by cooling to room temperature over 8 h. The result was yellow block-like crystals of **1** which were suitable for single-crystal X-ray diffraction (SCXRD) analysis. However, under the same reaction conditions, subsequent synthesis yielded only single crystals of **2**.

### 4.3. Synthesis of [Zn_2_(bpeb)_2_(dhbdc)_2_]·3DMA·H_2_O (2)   

A mixture of bpeb (5.6 mg, 0.02 mmol), H_2_dhbdc (4.0 mg, 0.02 mmol) and Zn(NO_3_)_2_·6H_2_O (12 mg, 0.04 mmol) dissolved in DMA (3.0 ml) and H_2_O (1.0 ml) was placed in a 20 ml glass tube. The tube was sealed and kept at 90–120°C for 48 h, followed by cooling to room temperature over 8 h. The resulting yellow block-shaped crystals were suitable for SCXRD analysis. Yield = 75%. Anal. Calcd [C_56_H_34_N_4_O_12_Zn_2_]: C, 59.57; H, 5.07; N, 7.15. Found: C, 59.21; H, 4.97; N, 7.02%. IR (KBr pellet, cm^−1^) 3444, 3264, 3030, 2929, 2877, 2781, 2584, 2475, 2287, 2072, 1942, 1844, 1757, 1641, 1608, 1483, 1537, 1371, 1291, 1229, 1202, 1178, 1109, 1066, 1016, 959, 907, 868, 834, 815, 786, 739, 665, 609, 555 and 472. ^1^H-NMR (300 MHz, ppm): 9.12 (*d*, 4H, pyridyl protons of bpeb), 8.43 (*d*, 4H, pyridyl protons of bpeb), 8.10 (*d*, 2H, olefinic protons of bpeb), 7.90 (*s*, 4H, phenyl protons of bpeb), 7.71 (*d*, 2H, olefinic protons of bpeb), 7.32 (*s*, 2H, phenyl protons of H_2_dhbdc). Detailed ^1^H-NMR data are given in Fig. S9.

### 4.4. Synthesis of [Zn(bpeb)(dhbdc)]0.5perylene·H_2_O (3)   

A mixture of bpeb (5.6 mg, 0.02 mmol), H_2_dhbdc (4.0 mg, 0.02 mmol), Zn(NO_3_)_2_·6H_2_O (12 mg, 0.04 mmol) and perylene (3.8 mg, 0.015 mmol) dissolved in DMA (3.0 ml) and H_2_O (1.0 ml) was placed in a 20 ml glass tube. The tube was sealed and kept at 90–120°C for 48 h, followed by cooling to room temperature over 8 h. The resulting orange block-shaped crystals were suitable for SCXRD analysis. Yield = 60%. Anal. Calcd [C_38_H_26_N_2_O_6_Zn]: C, 67.02; H, 4.00; N, 4.11. Found: C, 66.89; H, 4.13; N, 4.30%. IR (KBr pellet, cm^−1^) 3447, 3033, 2925, 2840, 2359, 2071, 1942, 1800, 1698, 1484, 1437, 1419, 1384, 1354, 1245, 1207, 1107, 1067, 1029, 962, 909, 866, 816, 788, 666, 601, 551 and 452. ^1^H NMR (300 MHz, ppm): 8.95 (*d*, 4H, pyridyl protons of bpeb), 8.43 (*d*, 4H, phenyl protons of perylene), 8.31 (*d*, 4H, pyridyl protons of bpeb), 8.12 (*d*, 2H, olefinic protons of bpeb), 7.95 (*s*, 4H, phenyl protons of bpeb), 7.86 (*d*, 4H, phenyl protons of perylene), 7.75 (*d*, 2H, olefinic protons of bpeb), 7.60 (*t*, 2H, phenyl protons of perylene), 7.34 (*s*, 2H, phenyl protons of H_2_dhbdc). Detailed ^1^H-NMR data are given in Fig. S10.

### 4.5. X-ray crystallographic analysis   

Crystal data for **1** at 173 K were collected on a Bruker SMART APEX II LTRA diffractometer equipped with graphite monochromated Mo *K*α radiation (λ = 0.71073 Å) generated by a rotating anode. Crystal data for **2** and **3** at 100 K were collected on a four-circle goniometer Kappa geometry Bruker AXS D8 Venture single-crystal X-ray diffractometer equipped with a Photon 100 CMOS active pixel sensor detector with Cu *K*α radiation (λ = 1.54178 Å). Data collection, data reduction and absorption correction were carried out using the software packages *APEX2* (Bruker, 2009 ▸) and *APEX3* (Bruker, 2016 ▸). All calculations for the structure determination were carried out using the *SHELXTL* package (Sheldrick, 2008 ▸). Relevant crystal, collection and refinement data for the crystal structures of **1**–**3** are summarized in Table S1. The CCDC numbers are 1955177–1955179 for **1**–**3**.

## 3. Results and discussion   

When bdc is replaced with 2,5-dihydroxy-1,4-benzene dicarboxylate (dhbdc) in the synthesis, in our first attempt we isolated single crystals of [Zn(bpeb)(dhbdc)] (**1**) with **dia** topology. Unfortunately, we could not isolate this MOF again in the subsequent synthesis. Instead, we obtained its supramolecular isomer [Zn_2_(bpeb)_2_(dhbdc)_2_] (**2**), which has a double-pillared-layer structure. However, when perylene was used as an additive, the ‘disappeared supramolecular isomer’ **dia** serendipitously reappeared with the inclusion of the perylene in the voids as [Zn(bpeb)(dhbdc)]·0.5perylene (**3**) (*i.e.* 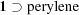). Details of these results are given below.

Yellow block crystals of **1** suitable for SCXRD data collection were obtained under solvothermal conditions from Zn(NO_3_)_2_·6H_2_O, 2-5-dihydroxy-1,4-benzenedicarboxylicacid (H_2_dhbdc) and bpeb in a mixture of dimethylacetamide (DMA) and water at 90°C for 48 h followed by slow cooling. As mentioned previously, subsequent attempts to reproduce the synthesis of **1** under the same experimental conditions persistently resulted in yellow block crystals of **2**. In one of our attempts, we added perylene to the synthesis of **2** and single crystals of **3** were obtained. The purity of the bulk of **2** and **3** was confirmed by comparing the simulated PXRD patterns from the SCXRD data with the bulk samples (Figs. S1 and S2 of the supporting information).

**Figure 2:**
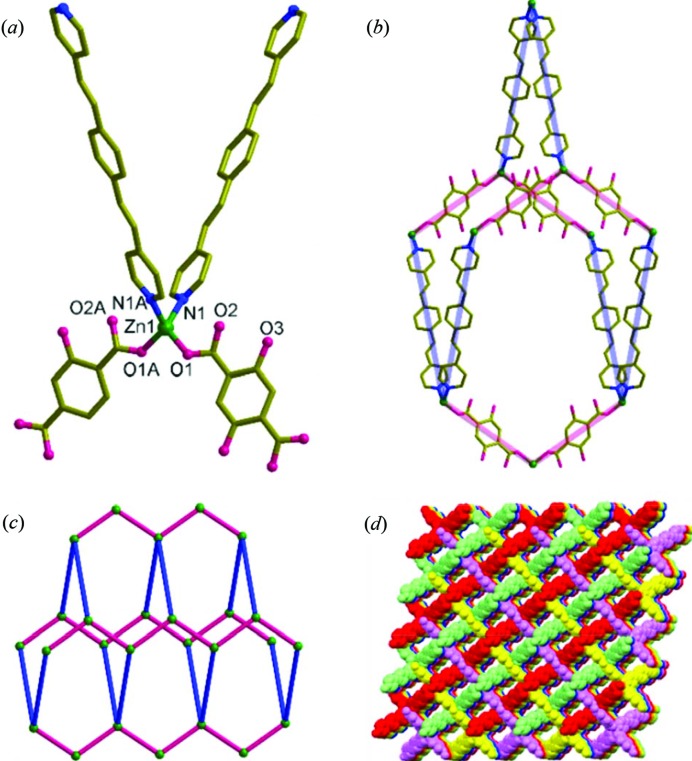
Structural details of **1**. (*a*) Coordination environment of Zn1. Symmetry code for A: 1.5 − *x*, *y*, 1.5 − *z*. (*b*) Single **dia** unit. (*c*) View of the **dia** topology. (*d*) Packing structure of the sixfold interpenetration shown along the *b* axis.

SCXRD experiments carried out at −100°C revealed that **1** crystallized in the monoclinic space group *P*2*/n* with *Z* = 2. The asymmetric unit contains half the formula unit, in which Zn1 sits on the crystallographic twofold axis. Zn1 with a highly distorted tetrahedral geometry [95.6 (1)–123.6 (1)°] is coordinated to two nitrogen atoms of the bpeb spacer ligands and two oxygen atoms of the dhbdc ligands which are disordered [Fig. 2 ▸(*a*) and S5]. The Zn1⋯O2 distance of 2.98 Å indicates that the carbonyl oxygen atom is non-bonded. The two hydrogen atoms of the hydroxyl groups are intramolecularly hydrogen-bonded to the non-bonded carbonyl oxygen atoms of the dhbdc ligand. The crystallographic inversion present in the middle of each spacer ligand generates a 3D CP with **dia** topology as shown in Figs. 2 ▸(*b*) and 2 ▸(*c*). The large void produced in this connectivity is filled by sixfold interpenetration [Fig. 2 ▸(*d*)]. Despite this sixfold interpenetration, **1** has porous channels parallel to the *b* axis with a solvent accessible void of 25.8% calculated using *PLATON* (Spek, 2003 ▸), which is less than 30.8% observed for the corresponding MOF with the bdc ligand (Park *et al.*, 2014*b* ▸). The solvents in these channels [Fig. 2 ▸(*d*)] were found to be severely disordered water and a small fraction of DMA molecules.

**Figure 3:**
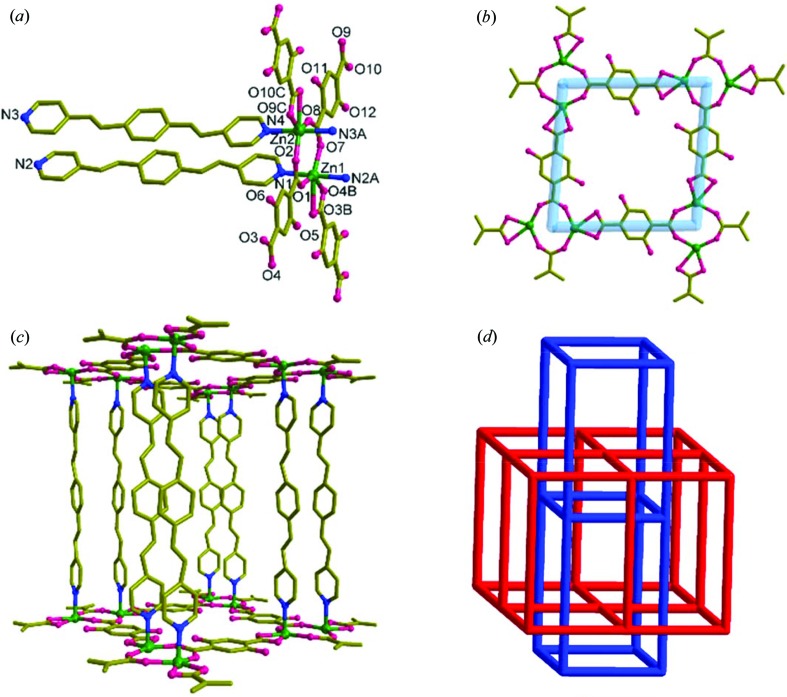
Structural details of **2**. (*a*) Coordination environments of Zn1 and Zn2. Symmetry operations: A: 1 + *x*, *y*, *z*; B: *x*, −*y*, −0.5 + *z*; C: *x*, 1 −*y*, 0.5 + *z*. (*b*) (4,4) grid showing the rhombus shape formed by Zn_2_(bdc)_2_. (*c*) View of the bpeb orientation and **pcu** topology. (*d*) Packing structure showing the twofold interpenetration. For clarity, the disorder and the hydrogen atoms are not shown.

The asymmetric unit of **2**, which crystallizes in *P*2/*c* with *Z* = 8, contains a formula unit with disordered bpeb ligands (Fig. S6). Interestingly, a bpeb ligand containing N1 and N2 atoms has both *trans*,*trans*,*trans* and *trans*,*cis*,*trans* conformations in the ratio 60 (1):40 (1), respectively, whereas the other bpeb ligand has only *trans*,*trans*,*trans* conformation in 68 (1):32 (1). The dinuclear repeating unit consists of two Zn(II) atoms bridged by two carboxylate groups and each Zn(II) is chelated by a carboxylate group [Fig. 3 ▸(*b*)]. The bridging carboxylates display the *syn*-*anti*-μ_2_-η^1^:η^1^ bonding mode as seen from two sets of Zn—O—C angles. The [Zn_2_(O_2_C-C)_2_] is roughly in a plane. The *exo*-carboxylate groups in the *para* positions of the dhbdc ligands are connected to generate a (4,4) layer structure of [Zn_2_(dhbdc)_2_] in the *bc* plane. In fact, the Zn—bpeb—Zn distance and the diagonal distances between the centres of the Zn_2_ dimer in the Zn_2_(dhbdc)_2_ rhomboidal ring are the *a* , *b* and *c* axes of the unit cell. Further examination reveals that each dhbdc has carboxylate bonding, incorporating both chelating and bridging modes (Park *et al.*, 2018 ▸). Due to the symmetrical bonding, the (4,4) grid has a rhombus shape with the dimensions 12.71 × 12.71 Å and an angle of 85.1°. The axial positions of the distorted octahedral Zn(II) centres are occupied by the nitrogen atoms of the bpeb ligands. The bpeb ligands act as pillars and connect the [Zn_2_(dhbdc)_2_] layers through pyridyl groups to produce double-pillared-layer structures [Fig. 2 ▸(*c*)]. The overall structure of **2** has a twofold *parallel* interpenetration as displayed in Fig. 2 ▸(*d*). The total potential solvent area volume as calculated by *PLATON* (Spek, 2003 ▸) in **2** is 2135.6 Å^3^, which is 32.9% of the unit-cell volume 6494.2 Å^3^.

**Figure 4:**
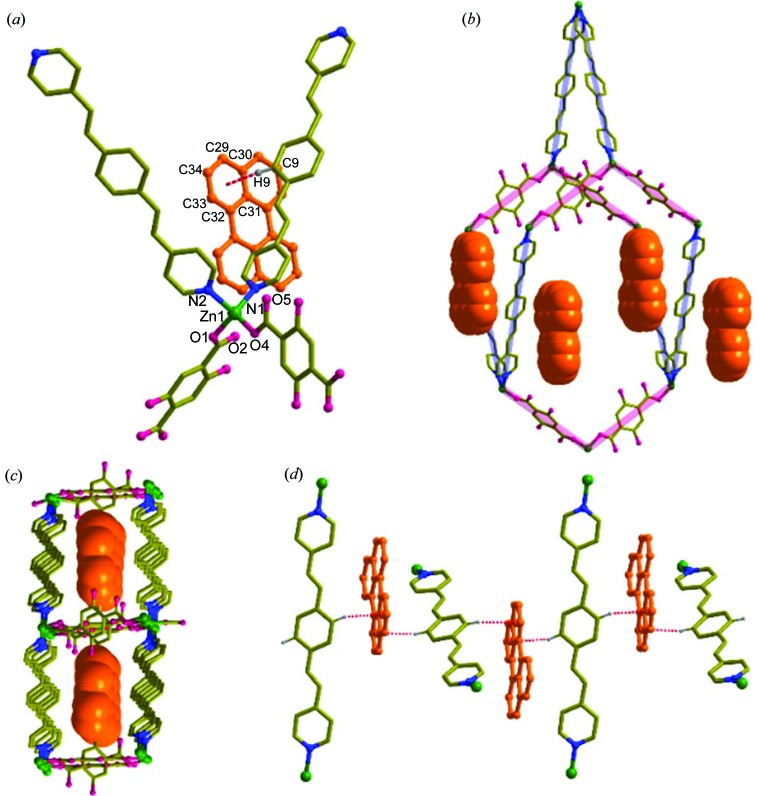
Structural details of **3**. (*a*) Coordination environment of Zn1 and the weak interaction of perylene with bpeb. (*b*) Single **dia** unit with perylene. (*c*) View of the channels along the *b* axis occupied by perylene. (*d*) Infinite pseudo-1D chain showing the C—H⋯π interactions between bpeb and perylene molecules.

Interestingly, **3** (*i.e.* 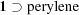) crystallizes in *C*2/*c* with *Z* = 8 and one formula unit in the asymmetric unit [Fig. 4 ▸(*a*)]. The structure is very similar to that of **1**, with sixfold interpenetrated **dia** network topology [Fig. 4 ▸(*b*)] and perylene is present in the channels along the *b* axis [Fig. 4 ▸(*c*)]. However, a detailed analysis by *TOPOS* (Blatov & Shevchenko, 2006 ▸) revealed that **1** belongs to Class Ia with *Z* = 6[6*1] and **3** belongs to Class IIIa with *Z* = 6[3*2] (where *Z* = *Z*_t_*Z_n_; *Z* is the total degree of interpenetration, *Z*_t_ is the translational degree of interpenetration and *Z*_n_ is the non-translational degree of interpenetration) (Blatov *et al.*, 2004 ▸). Further, a perylene guest also causes an increase in the crystallographic ordering of the bpeb ligands and a change in the unit cell. It is also noted that perylene interacts via C—H⋯π bonds with adjacent bpeb ligands [Figs. 4 ▸(*a*) and 4 ▸(*d*)]. We also tried the same synthesis with naphthalene, anthracene and pyrene, as substitutes for perylene, but these resulted in single crystals of only **2** without incorporation of these additives as confirmed by ^1^H-NMR spectra (Fig. S10). Perylene seems to be the best fit into the channel of **1**. Furthermore, we were not able to remove perylene from **3** to obtain **1**. Here, the kinetically unstable **dia** topology in **1** is stabilized by perylene in **3**.

Encapsulation of polyacenes in MOF materials is a simple yet powerful way of controlling the assemblies (Noh *et al.*, 2015 ▸; Stone & Anderson, 2007 ▸; Hashimoto *et al.*, 1998 ▸; Kitao *et al.*, 2017 ▸). The intermolecular π–π interactions with the hosts directly affect the optical and electronic properties of polyacenes. Hence, precise control of the assembly packing structures is also an effective strategy for tuning and enhancing their functions. Several approaches have been investigated to construct well controlled assemblies (Anthony *et al.*, 2001 ▸; Kitamura *et al.*, 2011 ▸; Kobayashi *et al.*, 2005 ▸; Mizobe *et al.*, 2009 ▸; Hinoue *et al.*, 2012 ▸; Kishi *et al.*, 2011 ▸; Prasad *et al.*, 2018 ▸).

**Figure 5:**
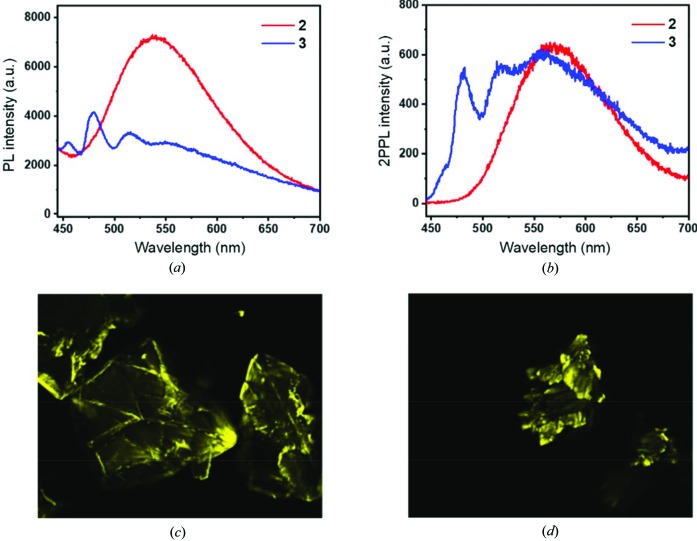
Solid-state (*a*) PL spectra and (*b*) 2PPL spectra of **2** and **3**. Confocal microscopic images of (*c*) **2** and (*d*) **3**. The colours, arbitrarily representative of the intensities, are measured by a photo-multiplier tube counter.

The photoluminescence (PL) spectra of **2** and **3** are shown in Fig. 5 ▸(*a*). The solid-state PL emission of **2** at λ_max_ = 539 nm (excitation at 400 nm) is similar to that of the double-pillared-layer MOF **2** [Fig. 1 ▸(*b*)] at λ_max_ = 530 nm (excitation at 365 nm) (Park *et al.*, 2018 ▸). Hence the influence of hydroxyl groups on the PL emission is very minimal although the dhbdc conformation in the [Zn(dhbdc)] chain is different (Fig. S7). It may be assumed that the PL emission of **1**, which cannot be measured, is also similar to that of the corresponding diamondoid MOF with the bdc ligand [Zn(bpeb)(bdc)] at λ_max_ = 495 nm (excitation at 365 nm) (Park *et al.*, 2014*a* ▸). Interestingly, the solid-state PL spectra of **3** has four peaks (455, 479, 514 and 550 nm) when excited at 400 nm. The three-finger pattern is that of perylene (at 455, 479 and 514 nm), with a broad peak around 550 nm that can be attributed to the overlap of **3** with the perylene emission. This can be understood from the poor host–guest interactions in **3** as shown in Fig. 4 ▸(*d*). Similar perylene-dominated emission has also been observed in other MOFs (Cui *et al.*, 2015 ▸; Chaudhari & Tan, 2018 ▸). Such emission from guest molecules was not observed when energy transfer occured via the Förster resonance energy transfer mechanism between the host MOFs and the guest molecules due to π–π interactions (Liu *et al.*, 2019 ▸, 2015 ▸; Quah *et al.*, 2015 ▸).

The 2PPL spectra of **2** and **3** are shown in Fig. 5 ▸(*b*). Figs. 5 ▸(*c*) and 5 ▸(*d*) show the bright images captured in the confocal microscope as a two-photon-excited process of **2** and **3**. The 2PPL emission of **2** at λ_max_ = 572 nm (excitation at 800 nm) is 33 nm red-shifted compared with the PL emission of **2**. The red-shift may be caused by the reabsorption effect (Yu *et al.*, 2013 ▸; Ren *et al.*, 2000 ▸; He *et al.*, 2008 ▸). The 2PPL emission of **3** agrees well with the PL spectrum [Fig. 5 ▸(*a*)]. The first hump peak at 455 nm of perylene in **3** is very weak and thus is observed to overlap with the peak at 480 nm. In particular, the peak at 577 nm of **3** is 27 nm red-shifted similar to **2**. As the sizes of the single crystals of **2** and **3** used in the measurements are different, the spectra are shown for the comparison of the peak positions rather than intensity.

## 4. Conclusions   

In summary, similar to the irreproducible ‘disappearing polymorphism’ which often frustrates organic solid-state scientists (Dunitz & Bernstein, 1995 ▸; Bernstein & Henck, 1998 ▸; Lancaster *et al.*, 2007 ▸, 2011 ▸; Bučar *et al.*, 2015 ▸), disappearing supramolecular isomerism has now been encountered serendipitously in the rapidly evolving field of CPs and MOFs. The MOF with **dia** topology could not be reproduced after its first isolation and subsequent attempts resulted in a double-pillared layer which is a supramolecular isomer. To our surprise, this **dia** topology reappeared when perylene was inadvertently added to the synthesis and included as a guest, 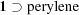. On the contrary, it is not clear why bdc readily forms the **dia** MOF while dhbdc does not. This is probably a new beginning for the realization of such phenomena in CPs and MOFs. Although the irreproducibility of this isomer can be attributed to the lack of insight into subtle experimental conditions (kinetic factors), these results nevertheless provide a new approach that could be utilized to recover not only a ‘supramolecular isomer’, but also polymorphs that have apparently ‘disappeared’.

## Supplementary Material

Crystal structure: contains datablock(s) 1, 2, 3. DOI: 10.1107/S2052252520001451/lq5029sup1.cif

Structure factors: contains datablock(s) 1. DOI: 10.1107/S2052252520001451/lq50291sup2.hkl

Structure factors: contains datablock(s) 2. DOI: 10.1107/S2052252520001451/lq50292sup3.hkl

Structure factors: contains datablock(s) 3. DOI: 10.1107/S2052252520001451/lq50293sup4.hkl

Supporting tables and figures. DOI: 10.1107/S2052252520001451/lq5029sup5.pdf

CCDC references: 1955177, 1955178, 1955179, 1955178, 1955179
