# Supplementary material for: Disappeared supramolecular isomer reappears with perylene guest
Source: IUCrJ. 2020 Feb 27;7(Pt 2):324–30. doi: 10.1107/S2052252520001451 (PMC7055374; doi:10.1107/S2052252520001451)
Supplement: Supplementary file 5 [file m-07-00324-sup5.pdf]

IUCrJ

Volume 7 (2020)

Supporting information for article:

Disappeared supramolecular isomer reappears with perylene guest

In-Hyeok Park, Atanu Dey, Kenta Sasaki, Masaaki Ohba, Shim Sung Lee and Jagadese J. Vittal

X-ray Crystallographic Analysis

Refinement Details of 1.

Our initial attempt to refine the structure did not yield satisfactory results. We were not able to locate the solvents. The final agreement factors were: R1 = 0.0485, wR2 = 0.1466 and Goof = 1.118 for 2534 reflections (I > 2σ). After squeezed, the final agreement factors are R1 = 0.0753, wR2 = 0.2414 and Goof = 1.110 for 2534 reflections (I > 2σ). The solvent accessible volume is 399.4 Å^3. The dhbdc is disordered. Two disordered components were refined with a common occupancy factor 0.514(4).

Hydrogen bond table:

D-H	d(D-H)	d(H..A)	<DHA	d(D..A)	A
C5-H5	0.930	2.481	134.83	3.205	O2a
C5-H5	0.930	2.657	177.82	3.586	O3a [-x+2, -y+1, -z+2]
O3a-H3a	0.820	1.864	145.92	2.584	O2a
O3a-H3a	0.820	2.244	146.79	2.964	O3a [-x+2, -y+1, -z+2]
O3Ab-H3Ab	0.820	1.792	146.51	2.516	O2A_b

Refinement Details of 2.

Total Potential Solvent Accessible Void Vol, 2135.6 Å^3. Our earlier attempts to locate and refine the solvent atoms were not yielded satisfactory results. Hence we resorted to SQUEEZE program and squeezed out the electron densities from the solvent region. In the main structure, two bpeb ligands were disordered. In one bpeb ligand C6-C15 atoms were disordered. In the second bpeb, N3-C35 atoms were disordered. Common occupancy factors were refined for each disorder to 0.683(13) and 0.601(12) respectively. Only isotropric thermal parameters could be refined for the non-hydrogen atoms in the disordered components.

Refinement Details of 3.

The structure refined well. Anisotropic thermal parameters were refined for all the non-hydrogen atoms. The electron densities at the final Fourier difference yielded 0.66 to -0.38 e/Å^3.

Hydrogen bond Table:

D-H	d(D-H)	d(H..A)	<DHA	d(D..A)	A
O3-H3	0.840	1.818	146.03	2.558	O2
O6-H6	0.840	1.833	146.09	2.574	O5
C1-H1	0.950	2.577	165.78	3.506	O6 [x, -y+1, z+1/2]
C5-H5	0.950	2.478	123.47	3.105	O5
C11-H11	0.950	2.551	118.39	3.116	O1

Table S1. Crystallographic data and refinement parameters of **1-3**

	**1**	**2**	**3**
formula	$C_{28}H_{20}N_2O_6Zn$	$C_{56}H_{40}N_4O_{12}Zn_2$	$C_{38}H_{24}N_2O_6Zn$
formula weight	545.83	1091.66	669.96
crystal system	Monoclinic	Monoclinic	Monoclinic
space group	*P*2/*n*	*P*2/*c*	*C*2/c
a (Å)	14.2399(5)	20.1826(9)	28.180(4)
b (Å)	7.5126(3)	18.7307(7)	14.880(2)
c (Å)	14.7479(5)	17.1854(6)	18.158(3)
α (deg)	90	90	90
β (deg)	101.6192(19)	91.583(2)	124.421(6)
γ (deg)	90	90	90
V (Å^3)	1545.38(9)	6494.2(4)	6280.7(17)
Z	2	4	8
D_{calc}(g/cm^3)	1.173	1.117	1.421
μ (mm^{-1})	0.832	1.354	1.515
$2\theta_{max}$ (deg)	51.998	133.698	135.358
reflections collected	18498	49531	24805
independent reflections	2543 [R_{int}=0.0367]	7776 [R_{int}=0.0670]	4436 [R_{int}=0.0558]
goodness-of-fit on F^2	1.118	1.091	1.032
R_1, wR_2 [I> 2σ(I)]	0.0485, 0.1466	0.0768, 0.2231	0.0558, 0.1250
R_1, wR_2 (all data)	0.0577, 0.1522	0.0997, 0.2401	0.0874, 0.1387

The CCDC numbers are 1955177-1955179 for **1**-**3**.

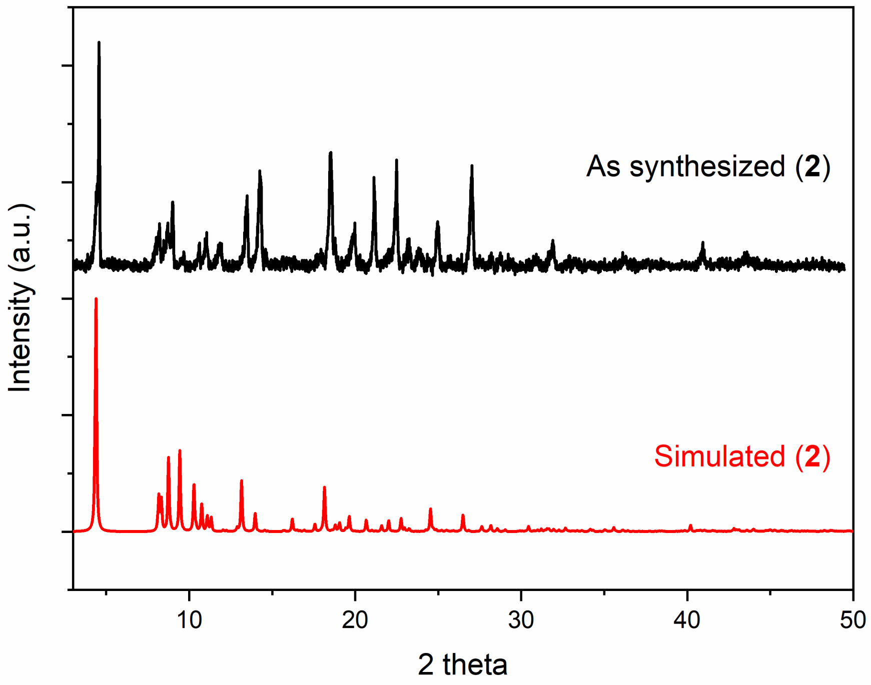

Figure S1 PXRD patterns of **2** (top) as synthesized and (bottom) simulated from the single crystal X-ray diffraction data.

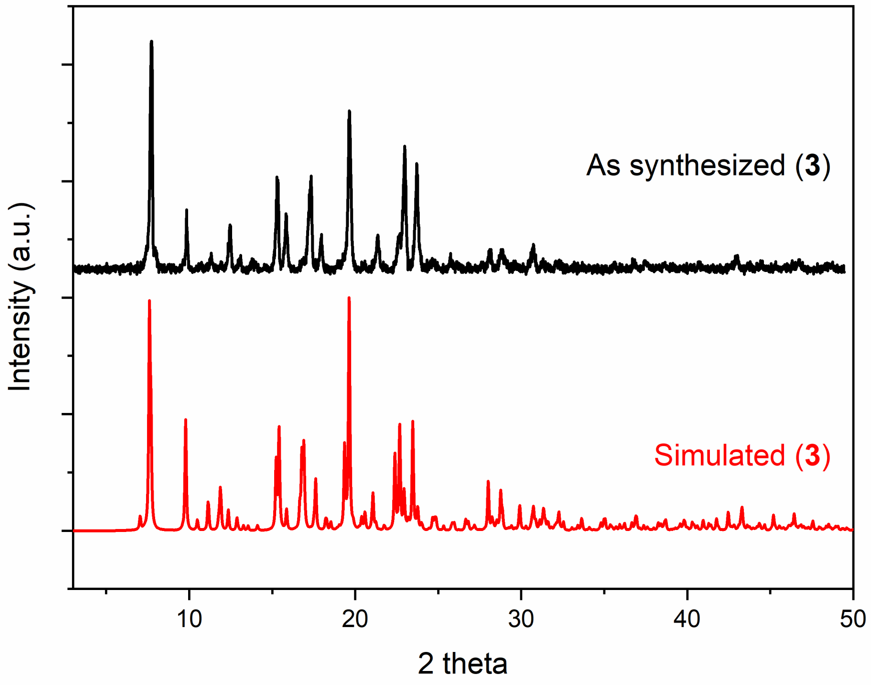

Figure S2 PXRD patterns of **3**: (top) as synthesized and (bottom) simulated from the single crystal X-ray diffraction data.

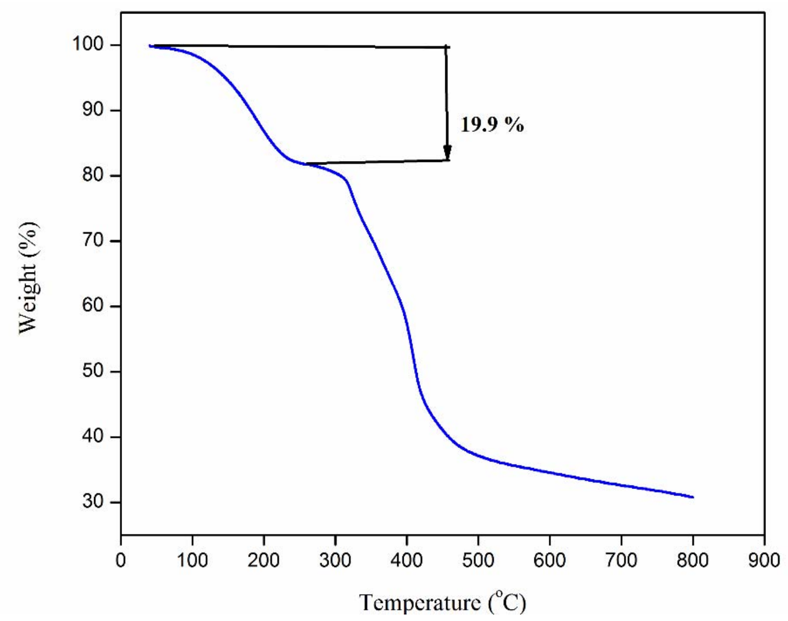

Figure S3 TGA curve of **2** with heating rate of 5 °C·min^{-1} under N_2 flow.

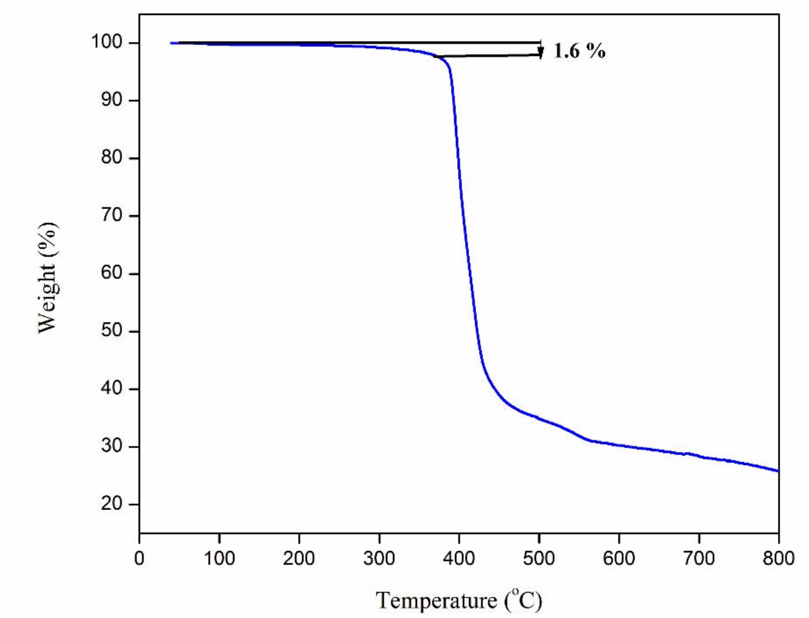

Figure S4 TGA curve of **3** with heating rate of 5 °C·min^{-1} under N_2 flow. **3** is stable up to 350 °C.

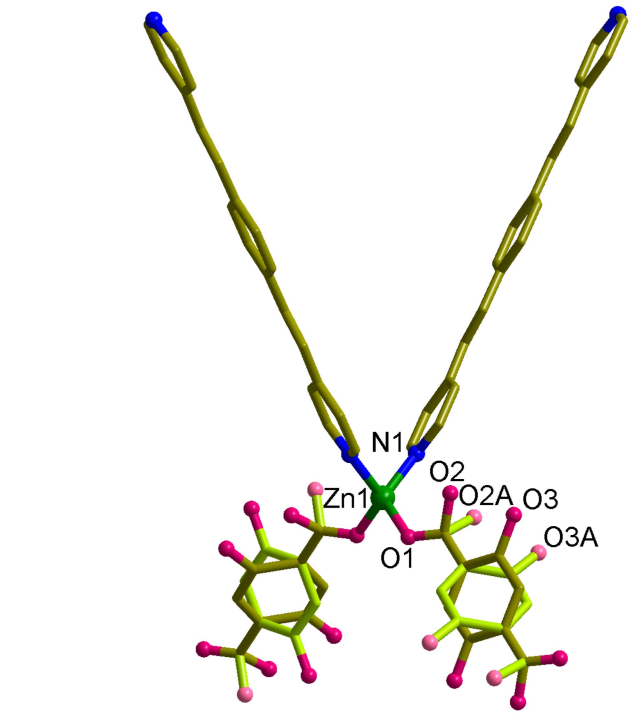

Figure S5 The disordered dhbdc ligands in **1**.

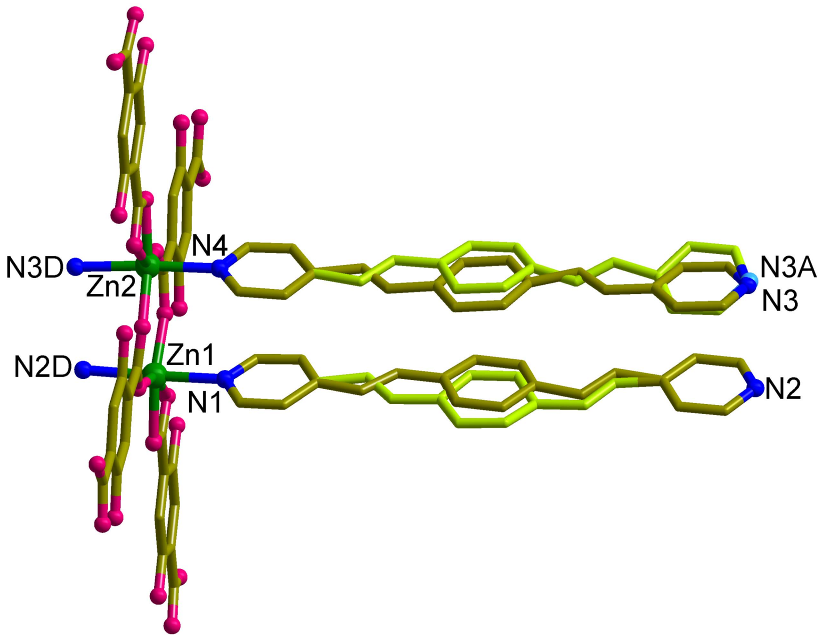

Figure S6 The disordered bpeb ligands in **2**. Symmetric units for A: 1+x, y, z; D: 1+x, y, z.

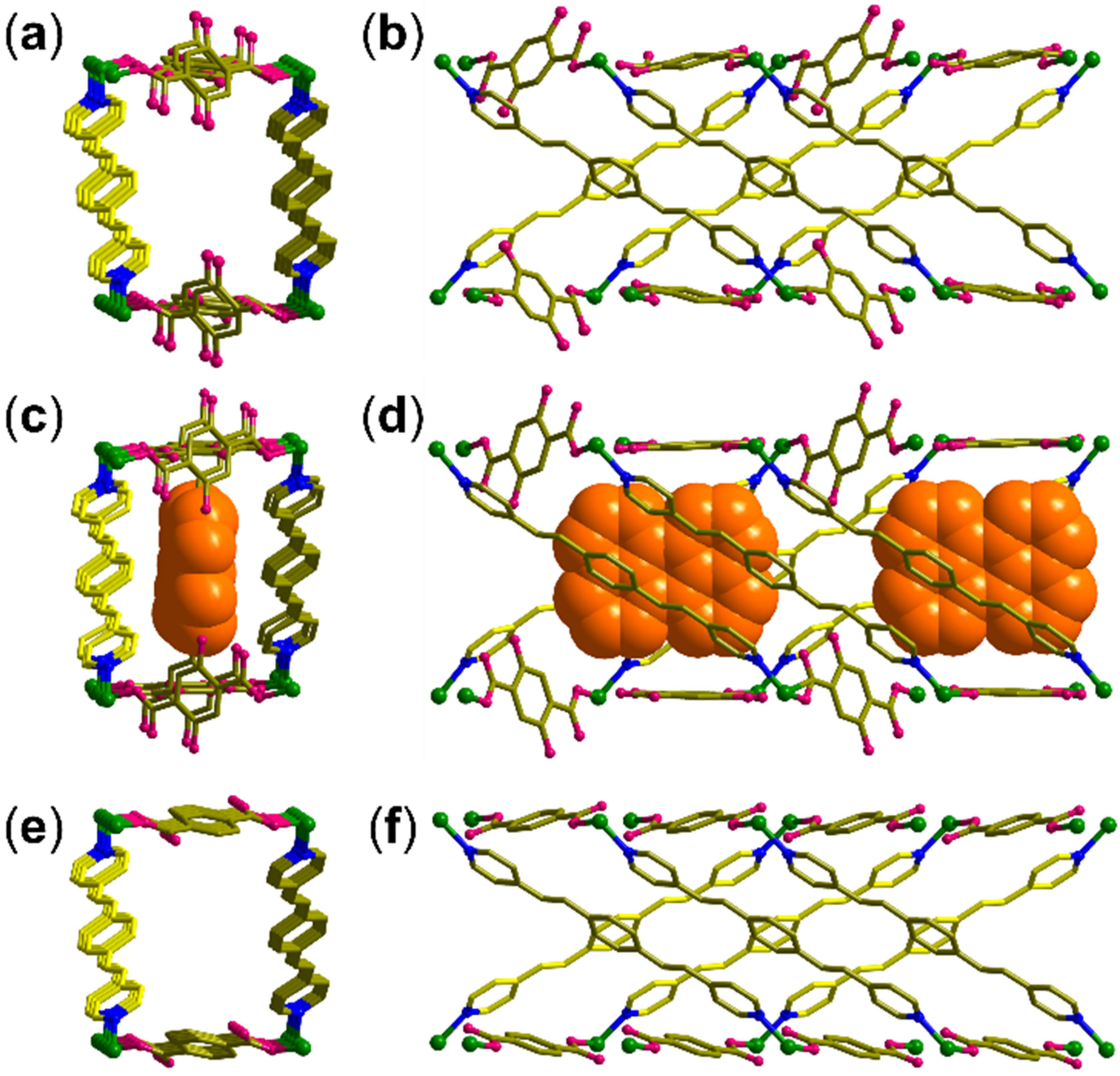

Figure S7 Structural comparison of dhbdc angles in (a, b) **1,** (c, d) **3**, and (e, f) **4**.

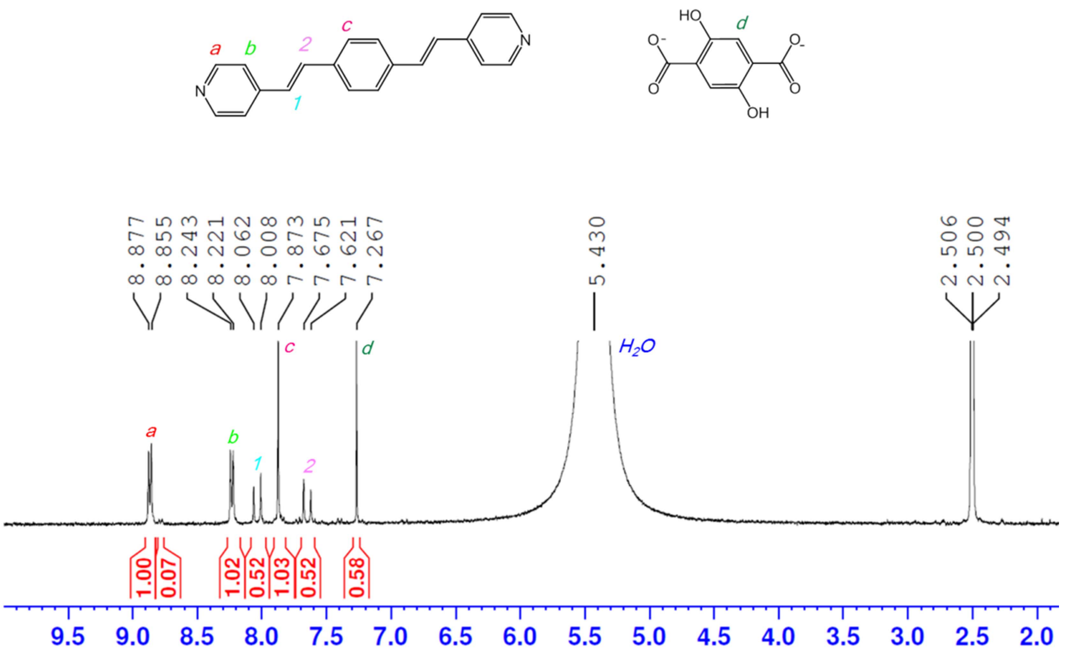

Figure S8 ^{1}H NMR spectrum of **2** in DMSO-d_6 with a small drop of HNO_3 to dissolve the crystals. The humps around 5.4 ppm is due to the protonated water.

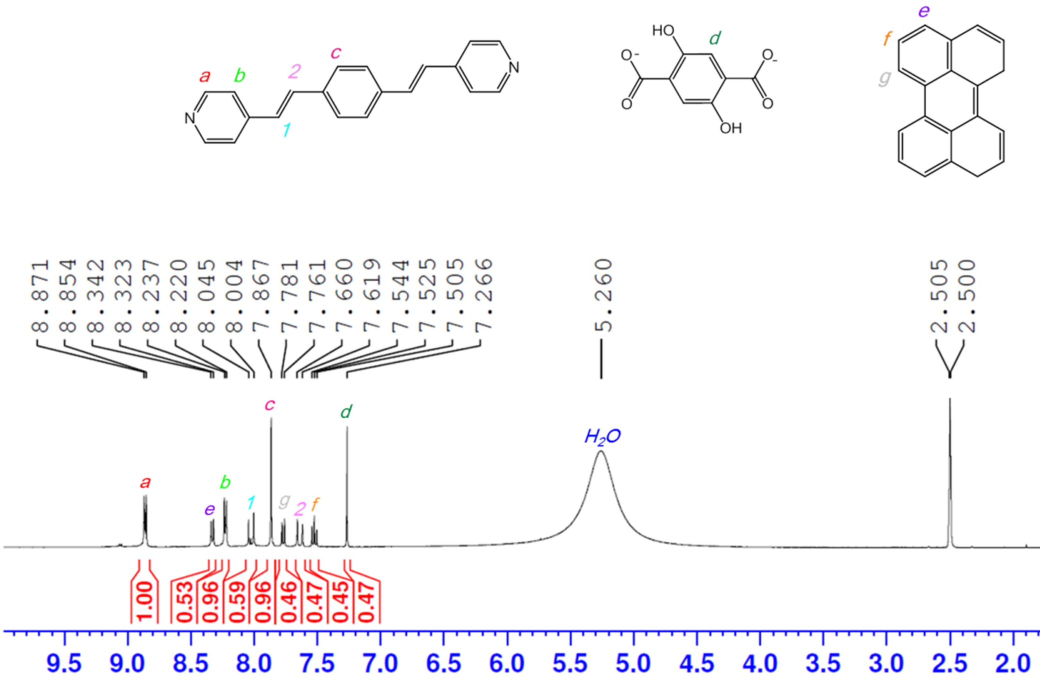

Figure S9 ^{1}H NMR spectrum of **3** in DMSO-d_6 with a small drop of HNO_3 to dissolve the crystals. The humps around 5.2 ppm is due to the protonated water.

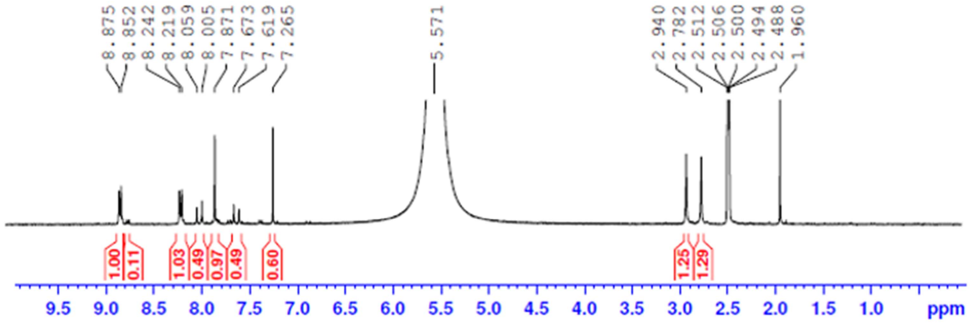

(a)

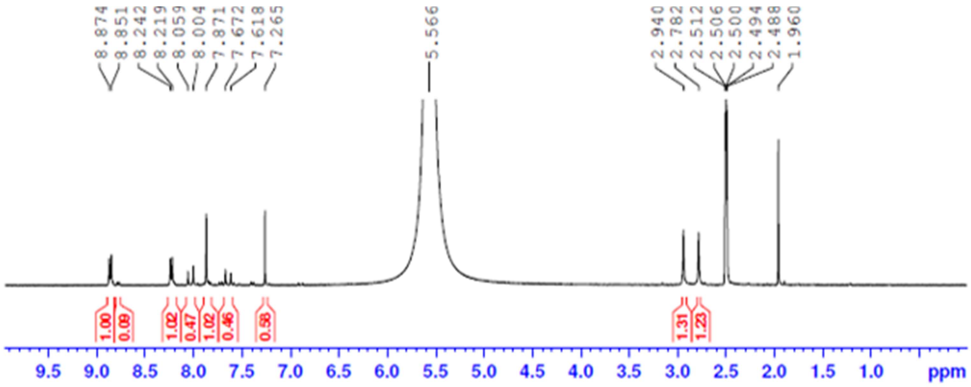

(b)

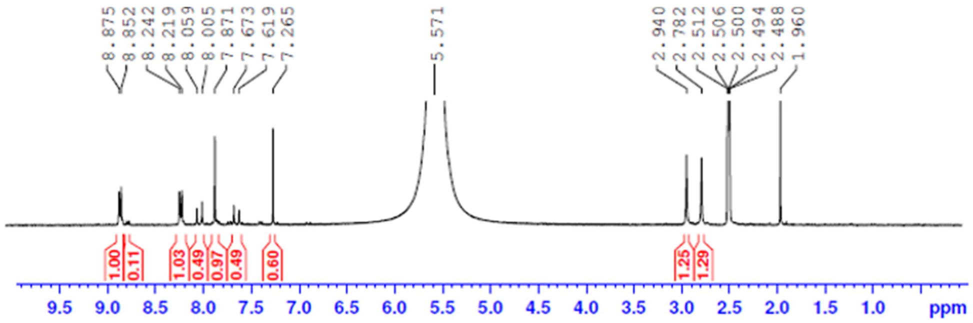

(c)

Figure S10 ^{1}H NMR spectra of single crystals from the reactions with presence of (a) naphthalene, (b) anthracene, and (c) pyrene (instead of perylene) in DMSO-d_6 with a small drop of HNO_3 to dissolve the crystals.

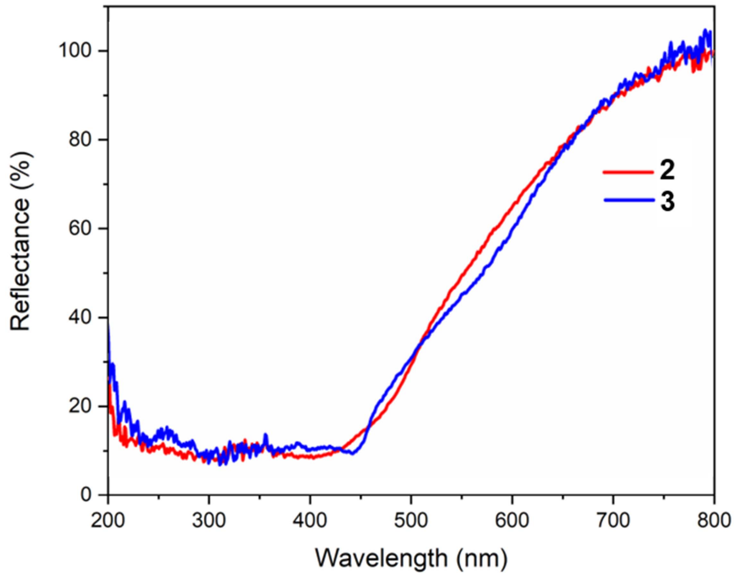

Figure S11 Solid-state reflectance UV of **2** and **3**.

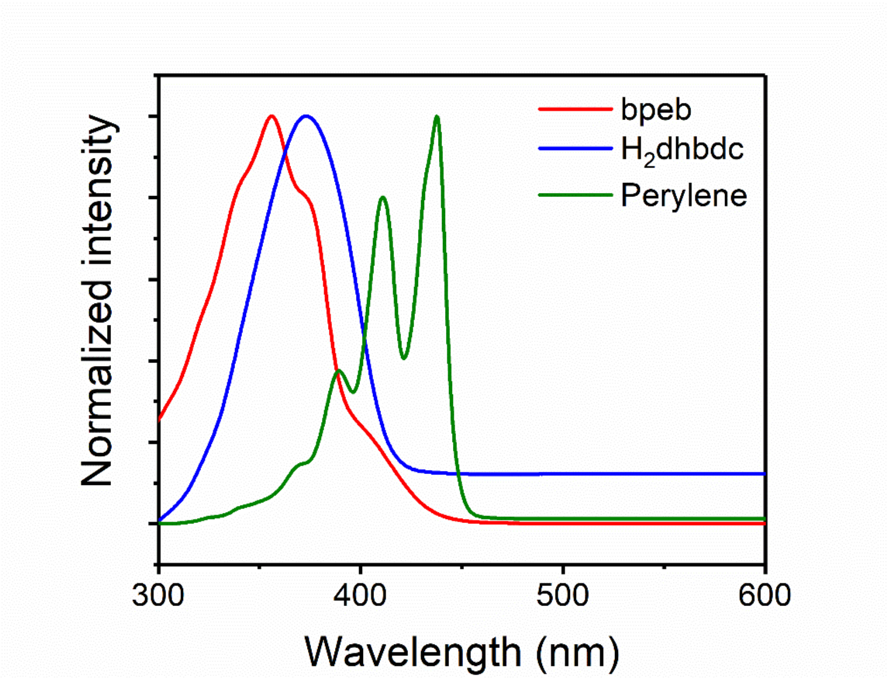

Figure S12 Solution UV-vis absorption spectra of bpeb, H_2dhbdc, and perylene in DMF.

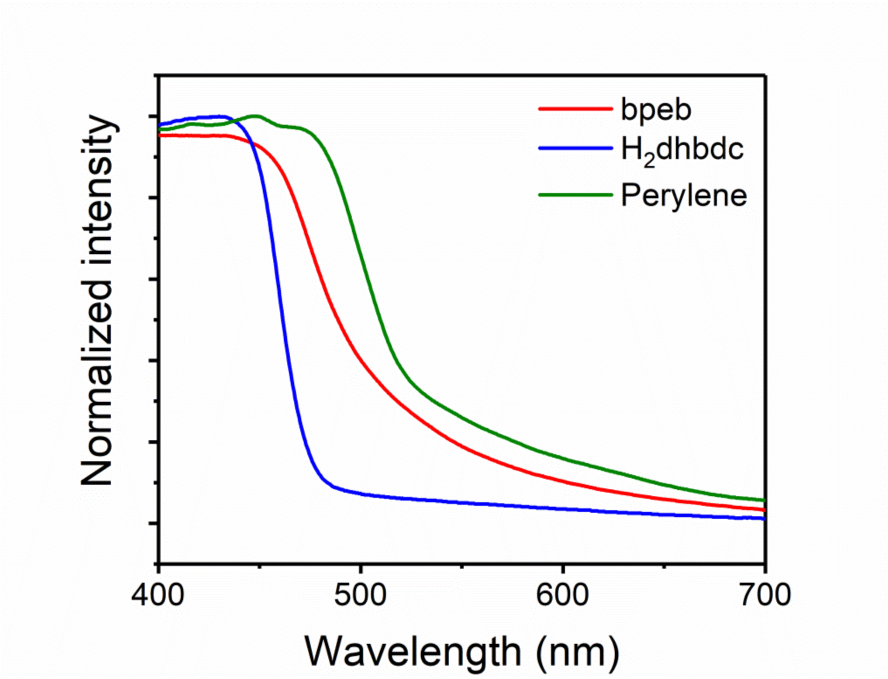

Figure S13 Solid-state UV-vis absorption spectra of bpeb, H_2dhbdc, and perylene.

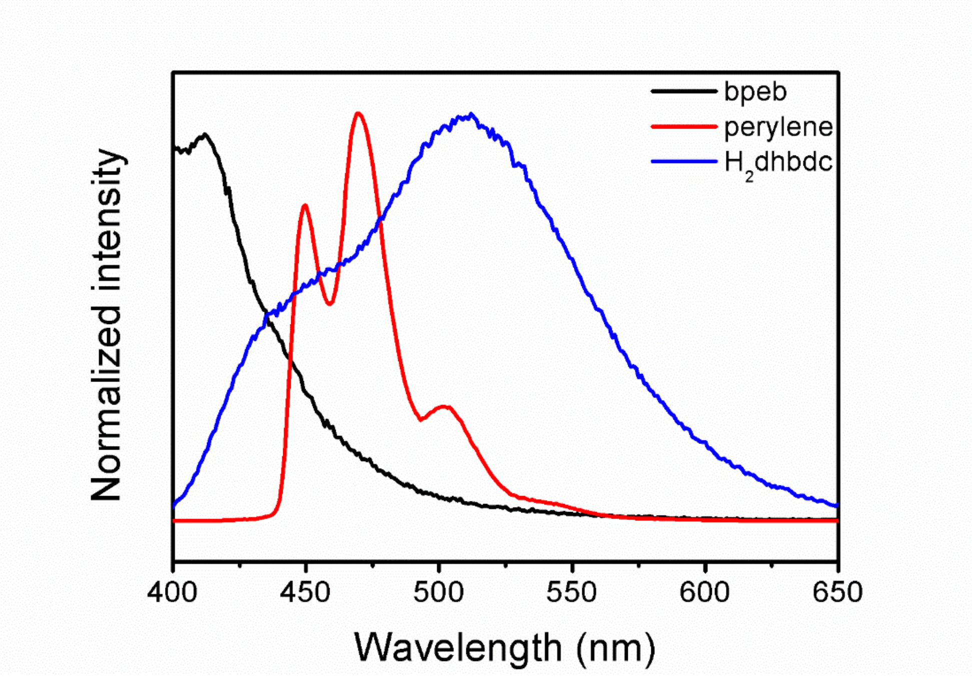

Figure S14 Solution PL spectra of bpeb, H_2dhbdc, and perylene.

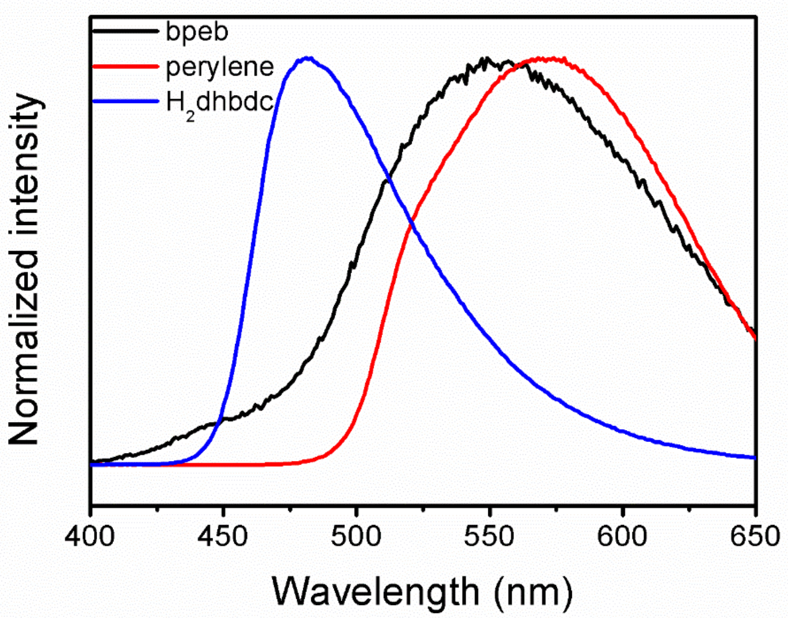

Figure S15 Solid-state PL spectra of bpeb, H_2dhbdc, and perylene.
